# Chromogranin B Protects Human Umbilical Endothelial Cells against Oxidative Stress

**DOI:** 10.3390/ijms251910296

**Published:** 2024-09-25

**Authors:** Elena Grossini, Sakthipriyan Venkatesan, Mohammad Mostafa Ola Pour, Daniela Ferrante, Daniela Surico, Rosanna Vaschetto, Vincenzo Cantaluppi, Mario Pirisi

**Affiliations:** 1 Laboratory of Physiology, Department of Translational Medicine, Università del Piemonte Orientale, 28100 Novara, Italy; sakthipriyan.venkatesan@uniupo.it (S.V.); 20046522@studenti.uniupo.it (M.M.O.P.); 2 Statistical Unit, Department of Translational Medicine, Università del Piemonte Orientale, 28100 Novara, Italy; daniela.ferrante@med.uniupo.it; 3 Gynecology and Obstetrics, Department of Translational Medicine, Università del Piemonte Orientale, 28100 Novara, Italy; daniela.surico@med.uniupo.it; 4 Anesthesia and Intensive Care, Department of Translational Medicine, Università del Piemonte Orientale, 28100 Novara, Italy; rosanna.vaschetto@med.uniupo.it; 5 Nephrology Unit, Department of Translational Medicine, Università del Piemonte Orientale, 28100 Novara, Italy; vincenzo.cantaluppi@med.uniupo.it; 6 Internal Medicine Unit, Department of Translational Medicine, Università del Piemonte Orientale, 28100 Novara, Italy; mario.pirisi@med.uniupo.it

**Keywords:** cardiovascular, granines, mitochondria, nitric oxide, oxidative stress, protection

## Abstract

Chromogranin B (CgB) is involved in the control of the cardiovascular system through the regulation of catecholamine release. Whether CgB can exert direct actions on the endothelium has not yet been clarified. Here, we aimed to investigate the effects of CgB on cell viability, mitochondrial membrane potential, reactive oxygen species (ROS), glutathione (GSH), nitric oxide (NO) release, and the cytosolic calcium concentration ([Ca^2+^]c) in human vascular endothelial cells (HUVECs) cultured under both physiological and peroxidative conditions. In HUVECs, experiments were conducted to establish the proper concentration and timing of CgB stimulation. Thereafter, specific assays were used to evaluate the response of HUVECs cultured in physiologic or oxidative stress conditions to CgB in the presence or absence of β-adrenergic receptor agonists and antagonists and intracellular pathways blockers. Analysis of cell viability, mitochondrial membrane potential, and NO release revealed that CgB was able to cause increased effects in HUVECs cultured in physiological conditions. Additionally, the same analyses performed in HUVECs cultured with H_2_O_2_, showed protective effects exerted by CgB, which was also able to counteract ROS release and maintain GSH levels. Furthermore, CgB played a dual role on the [Ca^2+^]c depending on the physiological or peroxidative cell culturing conditions. In conclusion, our data provide new information about the direct role of CgB in the physiological regulation of endothelial function and highlight its potential as a protective agent against peroxidative conditions, such as those found in cardiovascular diseases.

## 1. Introduction

Chromogranin B (CgB), also called secretogranin I, is a member of the granin family, along with chromogranin A (CgA) and secretogranin II (SgII) [1,2]. These proteins are proteolytically processed into active peptides that play regulatory roles in both physiological and pathophysiological contexts [3]. Regarding biological function, limited data suggest a role for CgB in the regulation of the cardiovascular system [4,5]. However, these suggestions are mostly based on indirect evidence from increased circulating levels of CgB in humans and animal models with altered myocardial conditions or during acute and chronic heart failure [6,7,8,9].

In the context of hypertension, it has been hypothesized that it could be related to augmented catecholamine release caused by CgB, potentially through activation of the autonomic nervous system. Even in this regard, there is a lack of clarity, as previous data also suggest a modulatory role for CgB on the hypertensive effects induced by epinephrine and norepinephrine [10,11].

What is particularly missing is information about the direct role of CgB on vascular function and its mechanisms of action. The only data available are related to the effects of an N-terminal peptide derived from CgB in microvascular models, which showed no intrinsic dilator response [12].

One possible target of CgB in endothelial cells, as a mechanism underlying its vascular effects, could be nitric oxide (NO). It is well known that changes in NO release can impair endothelial function and play a significant role in the pathophysiology of hypertension by increasing systemic vascular resistance [13,14,15]. In the presence of inflammation and oxidative stress, which are observed during hypertension, the production of NO in endothelial cells is drastically altered, causing NO to shift from being a protective factor to a harmful one [16,17]. In this regard, various isoforms of NO synthase (NOS) play a key role, with their activity regulated by changes in intracellular Ca^2+^ levels ([Ca^2+^]c) and intracellular pathways related to protein kinase B (Akt), extracellular signal-regulated kinases 1/2 (MEK1/2/ERK1/2), 5′ adenosine monophosphate-activated protein kinase (AMPK), Ca^2+^-calmodulin kinase (CaMKII), and protein kinase A (PKA) [18,19,20,21,22,23,24].

Another possible target of CgB in endothelial cells could be mitochondria and the redox balance, as these are widely reported to regulate vascular endothelial function and contribute to the onset of arterial hypertension [17,25].

Based on the above assumptions, a study aimed at examining the response of endothelial cells to CgB and its mechanisms of action could provide valuable data about the role of CgB in the pathophysiology of arterial hypertension and fill the knowledge gap on this issue.

For these reasons, the goal of this study was to evaluate the effects of CgB on cell viability, mitochondrial function, NO release, and [Ca^2+^]c in human umbilical vein endothelial cells (HUVECs) cultured under both physiological and peroxidative conditions. Additionally, we analyzed the involvement of Pi3K/Akt, MEK1/2/ERK1/2, AMPK, CaMKII, and PKA pathways in these effects.

## 2. Results

**Figure 1:**
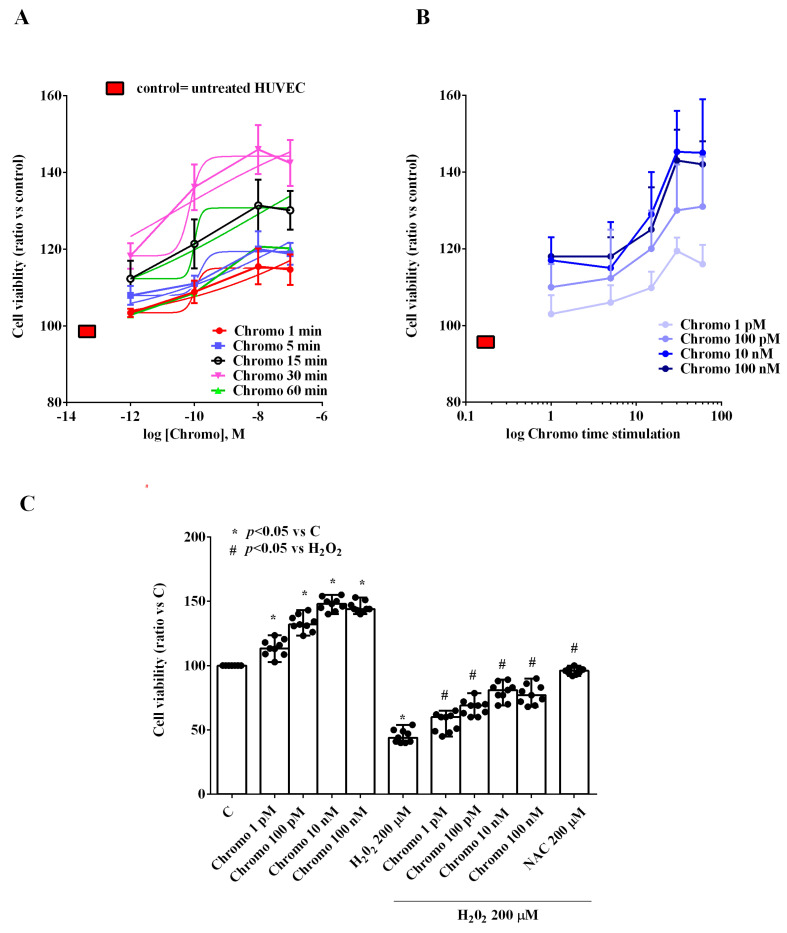
Effects of chromogranin B on cell viability of HUVECs. Panel (**A**) grading study; Panel (**B**) time-course study (time stimulation: 1 min, 5 min, 15 min, 30 min, and 60 min); Panel (**C**) effects of chromogranin B in the presence or absence of H_2_O_2_. The results are the medians and range of experiments repeated three times on different pools of HUVECs and triple readings. C = untreated HUVECs (DMSO only). Chromo = chromogranin B. NAC = N-acetylcysteine. In (**A**,**B**), the statistical analysis was performed by comparing the different chromogranin B concentrations at each stimulation time through the Kruskal–Wallis test, followed by Dunn’s post hoc test. In (**C**), the Mann–Whitney test was used to compare the different chromogranin B concentrations (“*” and “#” were considered together) to the controls and H_2_O_2,_ respectively. A *p*-value < 0.05 was chosen for statistical significance.

As shown in Figure 1, the grading and time-course study demonstrated that the cell viability of HUVECs cultured under physiological conditions increased in a dose-dependent manner when using CgB from 1 pM up to 10 nM, where a plateau was observed. Similarly, we observed that at 30 min of CgB stimulation, we could observe the maximum effect on cell viability (Figure 1A,B). Additionally, 30 min of CgB stimulation was able to counteract the effects of H_2_O_2_ on HUVECs in a dose-dependent manner, too (Figure 1C).

**Figure 2:**
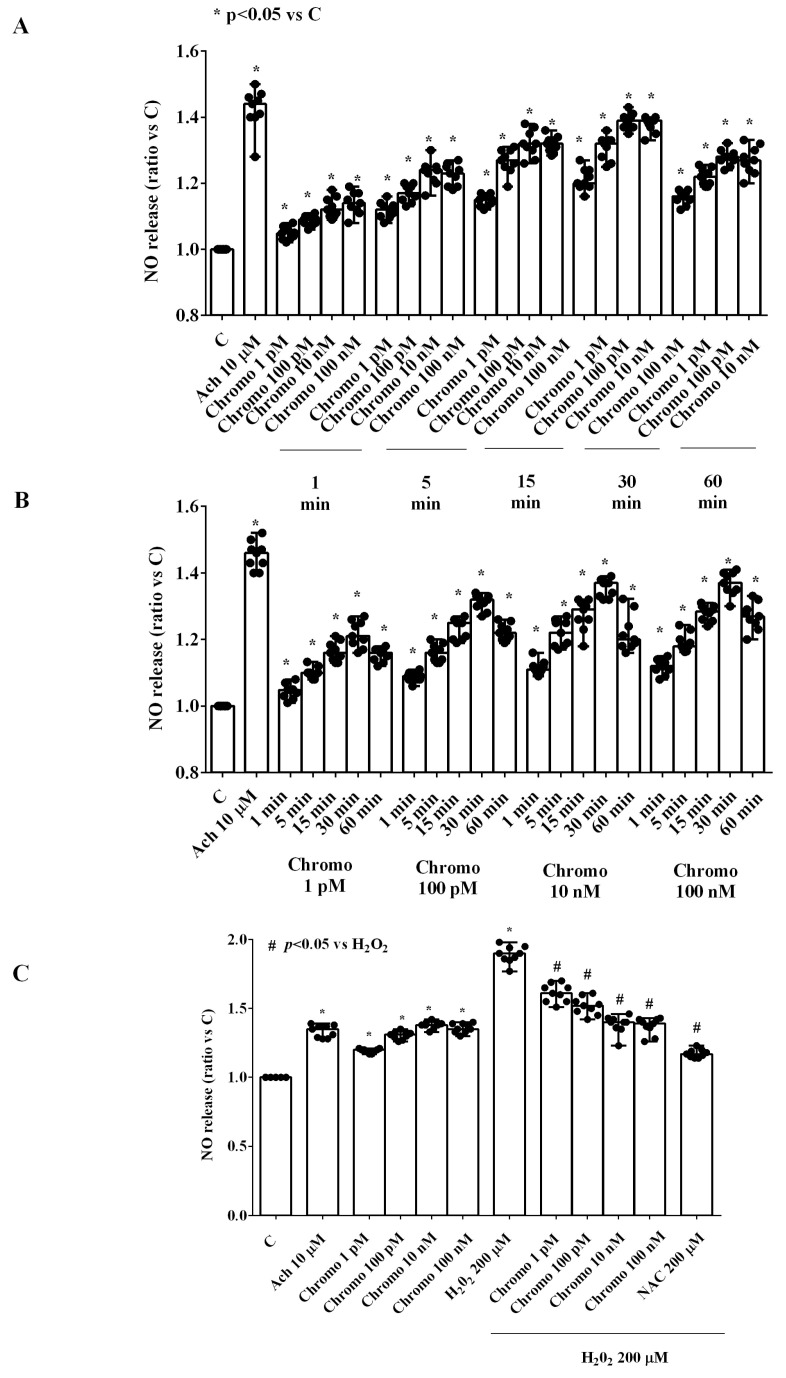
Effects of chromogranin B on nitric oxide (NO) release in HUVECs. Panel (**A**) grading study; Panel (**B**) time-course study; Panel (**C**) effects of chromogranin B in the presence or absence of H_2_O_2_. The results are the medians and range of experiments repeated three times on different pools of HUVECs and triple readings. Ach = acetylcholine. Other abbreviations are as in Figure 1. In (**A**,**B**), the statistical analysis was performed by comparing the different chromogranin B concentrations at each stimulation time through the Kruskal–Wallis test, followed by Dunn’s post hoc test. In (**C**), the Mann–Whitney test was used to compare the different chromogranin B concentrations (“*” and “#” were considered together) to controls and H_2_O_2_, respectively. A *p*-value < 0.05 was chosen for statistical significance.

The stimulation of HUVECs cultured under physiological conditions with CgB increased NO release in a dose-dependent manner (Figure 2). Similar to cell viability, the highest effect on NO release was observed at 10 nM of CgB. Additionally, the time-course study showed that 30 min of CgB stimulation resulted in the maximum NO release (Figure 2A).

Notably, in HUVECs cultured under peroxidative conditions, which was able to increase the release of NO, 30 min of CgB stimulation reduced NO release in a dose-dependent manner, up to 10 nM (Figure 2C).

**Figure 3:**
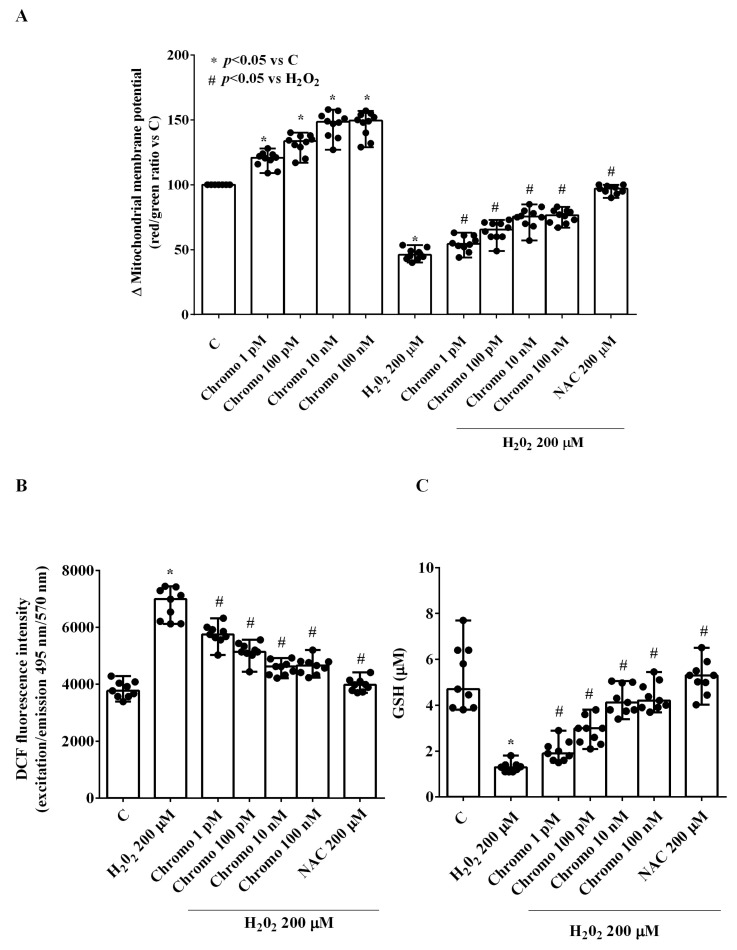
Effects of chromogranin B on mitochondrial membrane potential (**A**), reactive oxygen species release (**B**), and glutathione (**C**) in HUVECs. The reactive oxygen species release is shown as DCF fluorescence. The results are the medians and range of experiments repeated three times on different pools of HUVECs and triple readings. DCF = 2,7-dichlorodihydrofluorescein. GSH: glutathione. Other abbreviations are as in previous figures. In (**A**–**C**), the Mann–Whitney test was used to compare the different chromogranin B concentrations (“*” and “#” were considered together) to controls and H_2_O_2_, respectively. A *p*-value < 0.05 was chosen for statistical significance.

The protective effects elicited by CgB on HUVECs were confirmed by experiments measuring mitochondrial membrane potential. As shown in Figure 3A, in HUVECs cultured under physiological conditions, 30 min of CgB stimulation increased mitochondrial membrane potential in a dose-dependent manner, up to 10 nM. 

In the presence of H_2_O_2_, CgB was able to counteract the detrimental effects of the peroxidative stimulus. As shown in Figure 3B,C, CgB reduced the ROS release induced by H_2_O_2_ and increased glutathione (GSH) levels, which had been reduced by H_2_O_2_.

**Figure 4:**
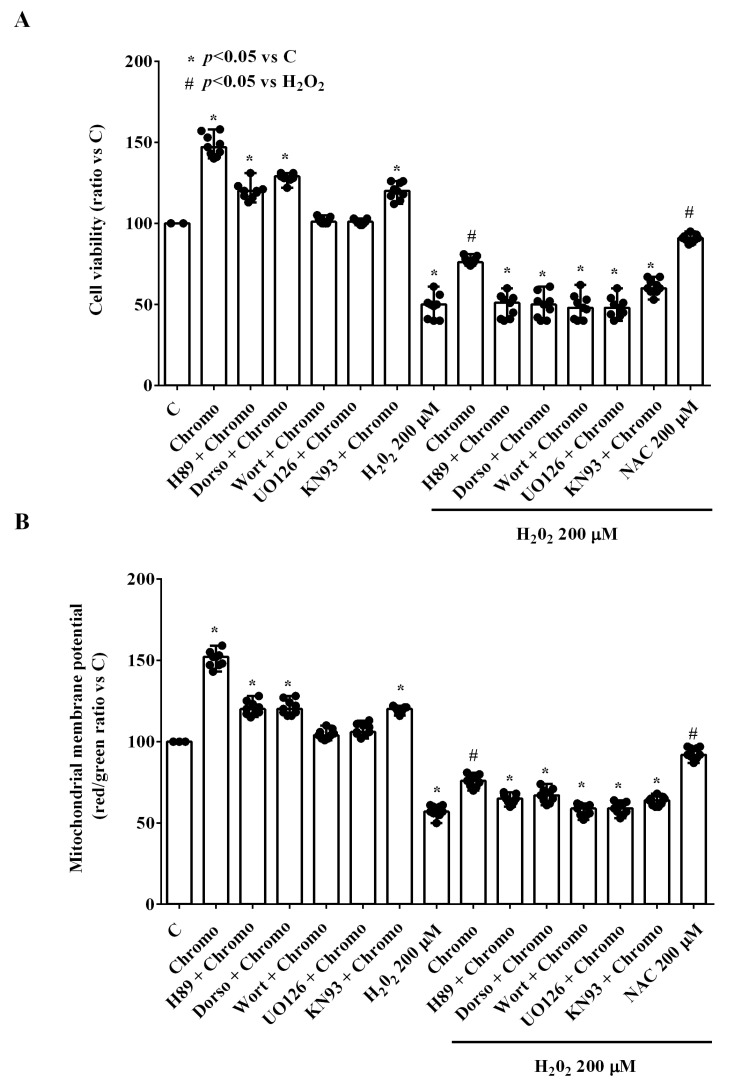
Effects of 10 nM of chromogranin B on cell viability (**A**) and mitochondrial membrane potential (**B**) in HUVECs in the presence or absence of various agonists/antagonists. The results are the medians and range of experiments repeated three times on different pools of HUVECs and triple readings. Dorso = dorsomorphin (10 nM). H89 = PKA inhibitor (10 nM). KN93 = CAMKII inhibitor (10 nM). UO126 = MEK1/2 inhibitor (10 nM). Wort = wortmannin (pan-PI3K inhibitor) (10 nM). Other abbreviations are as in previous figures. In (**A**,**B**), the Mann–Whitney test was used to compare the effects of chromogranin B with various blockers (“*” and “#” were considered together) to controls and H_2_O_2,_ respectively. A *p*-value < 0.05 was chosen for statistical significance.

To investigate the intracellular pathways involved in the effects of CgB on HUVECs cultured under physiological conditions, experiments were repeated in the presence of inhibitors for PKA, AMPK, pan-Pi3K, MEK1/2, and CaMKII: H89, dorsomorphin, wortmannin, U0126, and KN93 (10 nM), respectively. As shown in Figure 4A and Figure S1A, blocking the PKA, AMPK, and CaMKII pathways reduced the effects of CgB on cell viability in HUVECs cultured under physiological conditions. In the presence of ERK1/2 and Akt blockers, the effects of CgB were abolished (Figure 4A and Figure S1A). Similar results were observed for mitochondrial membrane potential (Figure 4B and Figure S1B).

In HUVECs cultured under peroxidative conditions, the PKA, AMPK, MEK1/2, and pan-Pi3K blockers prevented the protective effects of CgB on cell viability (Figure 4A and Figure S1A). In the presence of the CaMKII blocker, there was a reduction in these protective effects.

Regarding mitochondrial membrane potential, we observed that MEK1/2 and pan-Pi3K blockers were able to prevent the protective effects of CgB. In the presence of the PKA, AMPK, and CaMKII blockers, the effects of CgB were partially counteracted (Figure 4B and Figure S1B).

**Figure 5:**
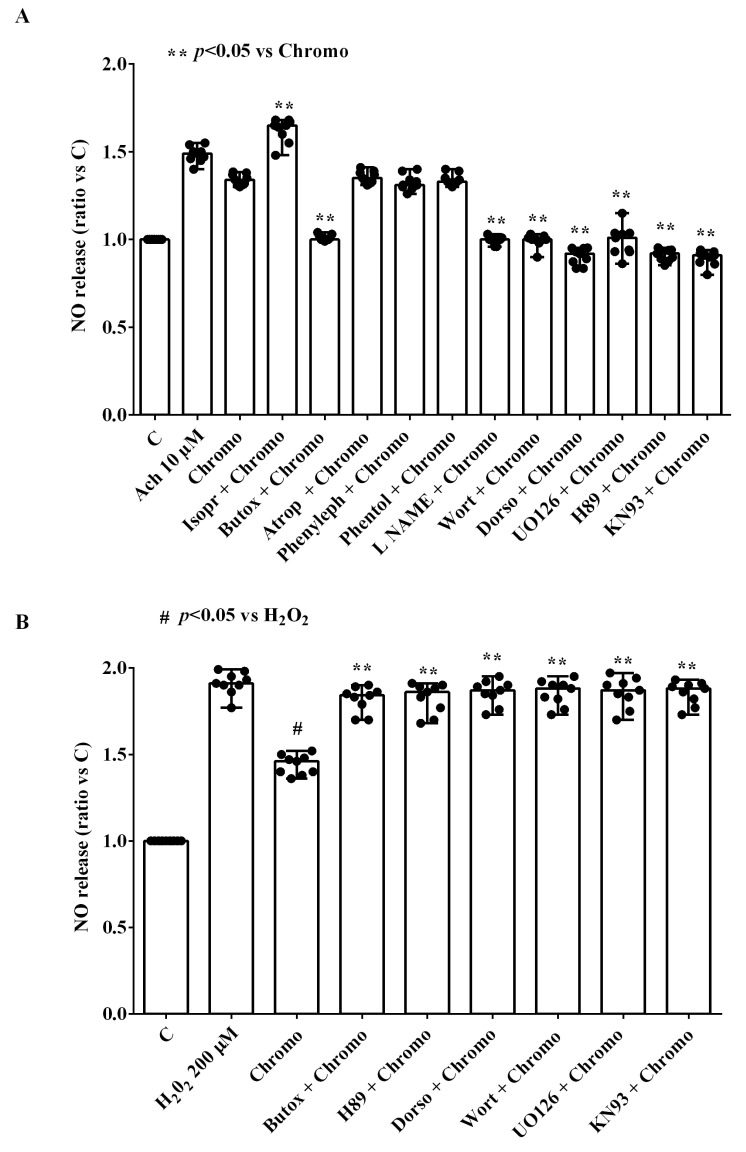
Effects of 10 nM chromogranin B on nitric oxide (NO) release in HUVECs in the presence or absence of various agonists/antagonists. The results are the medians and range of experiments repeated three times on different pools of HUVECs and triple readings. Atrop = atropine (cholinergic receptor inhibitor; 10 nM). Butox = butoxamine (β_2_-adrenergic receptor inhibitor; 10 nM). Isopr = isoproterenol (β-adrenergic receptor agonist; 10 nM). L-NAME = Nω-Nitro-L-arginine methyl ester hydrochloride (NO inhibitor; 10 mM). Phenyleph = phenylephrine (α-adrenergic receptor agonist; 10 nM). Phentol = phentolamine (α-adrenergic receptor antagonist; 10 nM). Other abbreviations are as in previous figures. In (**A**,**B**), the Mann–Whitney test was used to compare the effects of chromogranin B with various agonists/antagonists (“**” and “#” were considered together) to controls and H_2_O_2_, respectively. A *p*-value < 0.05 was chosen for statistical significance.

Regarding NO release, the effects of 30 min of CgB stimulation (10 nM) on HUVECs cultured under physiological conditions were potentiated by isoproterenol and abolished by butoxamine, dorsomorphin, wortmannin, U0126, H89, KN93 (all at 10 nM), and L-NAME (10 mM; Figure 5 and Figure S2). The cholinergic receptor agonist atropine, the α-adrenergic receptor agonist phenylephrine, and the α-adrenergic receptor antagonist phentolamine did not alter the response of HUVECs to CgB (Figure 5A and Figure S2A).

In HUVECs treated with H_2_O_2_, the effects of CgB were also counteracted by butoxamine, dorsomorphin, wortmannin, U0126, H89, and KN93 (all at 10 nM; Figure 5 and Figure S2).

**Figure 6:**
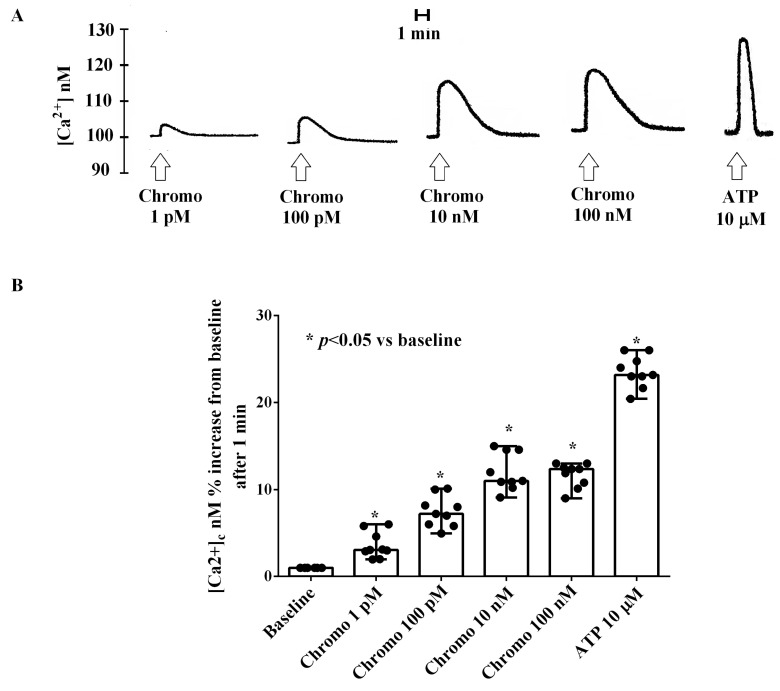
Effects of chromogranin B (1 pM, 100 pM, 10 nM, and 100 nM) on [Ca^2+^]c in HUVECs cultured in physiologic conditions. In (**A**), an example of traces is shown; in (**B**), results were obtained 1 min after the stimulation from three different experiments performed on different pools of HUVECs (and triple readings) as medians and range. Abbreviations are as in previous figures. In (**B**), the Mann–Whitney test was used to compare the different chromogranin B concentrations vs. baseline (“*” were considered together). A *p*-value < 0.05 was chosen for statistical significance.

As reported in Figure 6, CgB caused a dose-dependent and transient increase in the [Ca^2+^]c in HUVECs cultured under physiologic conditions. As depicted in Figure 6, the highest effect on the [Ca^2+^]c was observed with 10 nM of CgB. The increase in intracellular [Ca^2+^]c reached a peak approximately 1 min after CgB stimulation and returned to baseline after about 3 min (Figure 6A).

**Figure 7:**
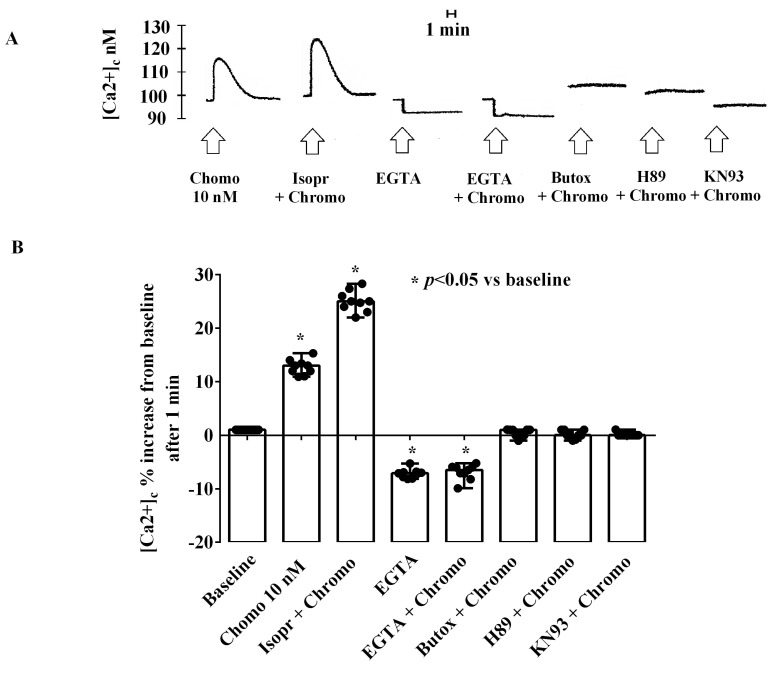
Effects of 10 nM of chromogranin B on [Ca^2+^]c in HUVECs cultured in physiological conditions in the presence or absence of various agonists/antagonists and ethylene glycol tetra-acetic acid (EGTA). In (**A**), an example of the traces is shown; in (**B**), results were obtained 1 min after stimulations from three different experiments performed on different pools of HUVECs (and triple readings) as medians and ranges. Chromo = chromogranin. Isopr = isoproterenol (10 nM). EGTA = ethylene glycol tetraacetic acid (50 mM). Butox = butoxamine (10 nM). H89 = PKA blocker (10 nM). KN93 = CAMKII inhibitor (10 nM). In (**B**), the Mann–Whitney test was used to compare the effects of chromogranin B with various agonists/antagonists and EGTA (“*”were considered together) vs. baseline. A *p*-value < 0.05 was chosen for statistical significance.

As shown in Figure 7 and Figure S3, the effects of 10 nM of CgB on the [Ca^2+^]c were almost abolished in HUVECs cultured in a Ca^2+^-free medium (50 mM of EGTA). In addition, they were potentiated by isoproterenol and abolished by butoxamine, H89, and KN93 (10 nM).

**Figure 8:**
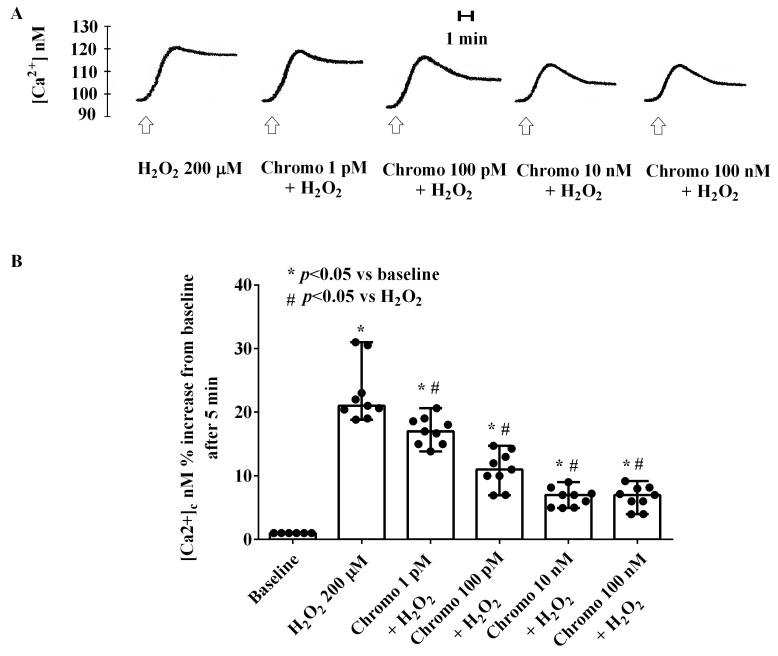
Effects of chromogranin B (1 pM, 100 pM, 10 nM, and 100 nM) on the [Ca^2+^]c in HUVECs cultured in peroxidative conditions. In (**A**), an example of the traces is shown; in (**B**), results were obtained 5 min after stimulations from three different experiments performed on different pools of HUVECs (and triple readings) as medians and range. Abbreviations are as in previous figures. In (**B**), the Mann–Whitney test was used to compare the different chromogranin B concentrations vs. baseline and H_2_O_2_ (“*”and ”#” were considered together)_,_ respectively. A *p*-value < 0.05 was chosen for statistical significance.

In addition, CgB was able to dose-dependently reduce the effects of H_2_O_2_ on the [Ca^2+^]c in HUVECs (Figure 8). As depicted in Figure 8, in the presence of CgB, H_2_O_2_ was still able to increase the [Ca^2+^]c but to a lesser extent and with a reduction in the plateau phase. Notably, the highest effects were observed in HUVECs stimulated with 10 nM of CgB.

The effects of CgB in HUVECs cultured under physiological and oxidative stress conditions are summarized in the graphical abstract.

The Graphical abstract shows the effects of CgB in HUVECs in physiological and oxidative stress conditions.

## 3. Discussion

The results of this study showed that CgB can improve cell viability, NO release, and mitochondrial function in HUVECs and can counteract the detrimental effects elicited by peroxidation. About this issue, we found that CgB was able to reduce the effects of H_2_O_2_ on NO and ROS release and prevent the decrease in GSH levels. In addition, CgB rapidly and transiently increased the [Ca^2+^]c in HUVECs cultured under physiological conditions while reducing the effects of H_2_O_2_. The effects on HUVECs were related to pathways involving β-adrenergic receptors, PKA, AMPK, MEK1/2/ERK1/2, Pi3K/Akt, and CaMKII.

Regarding CgA and CgA-derived peptides, there is ample information about their effects on a wide range of cells and tissues, such as the vascular endothelium, endocardium, cardiomyocytes, and vascular smooth muscle cells [26,27]. For instance, vasostatin I was found to cause relaxation of bovine arteries through the involvement of K+ channels and inhibit endothelial cell migration and capillary-like structure formation induced by vascular endothelial growth factor and basic fibroblast growth factor [1,28]. Additionally, the C-terminal sequence of vasostatin II, CgA79–113, was reported to inhibit the vasoconstrictive effects of angiotensin II, whereas catestatin-induced proliferation, migration, and in-vitro angiogenesis in human venous endothelial cells and improved limb ischemia by inducing angiogenesis, vasculogenesis, and arteriogenesis. The mechanisms underlying these effects were related to ERK and Pi3K pathways [29]. In general, vasostatins and catestatin were found to exert negative effects on cardiac function through mechanisms related to β-adrenergic receptor involvement and the activation of intracellular pathways related to Pi3K/AKT and NO. In contrast, the C terminal CgA peptide, serpinin, increased cardiac contractility and relaxation in the Langendorff-perfused rat heart and in papillary muscles through the involvement of β_1_-adrenergic receptors and the adenylate cyclase/PKA pathway [28,30].

In the case of CgB, which is the most abundant core protein in human catecholamine secretory vesicles, it is known to significantly influence their biogenesis and secretion, as well as the autonomic tone regulating arterial blood pressure [11]. Additionally, CgB plasma levels have been reported to act as an independent predictor of left ventricular functional recovery after percutaneous coronary intervention [30].

In spite of this, however, no data are currently available regarding the possible mechanisms of actions on CgB and, in particular, on the direct effects of CgB on endothelial function. While it is well known that CgB may play a role in the regulation of the cardiovascular system and the onset of hypertension through the release of catecholamines [31,32], little is known about its possible direct involvement.

An in-depth study of the role of CgB on the function of vascular endothelial cells could help better understand the mechanisms underlying its involvement in the genesis of cardiovascular disease, independent of catecholamine release regulation.

For these reasons, we investigated the effects of CgB on HUVECs cultured under both physiological and peroxidative conditions, focusing on cell viability, mitochondrial function, the oxidant/antioxidant system, and NO release, as well as the pathways involved.

In the preliminary phase, we evaluated the optimal concentration and stimulation time for CgB through a dose- and time-response study performed on cell viability and mitochondrial membrane potential. The concentrations of CgB used were similar to those found in the plasma of normal subjects and patients with respiratory failure, heart failure, angina, and neuroendocrine tumors [33,34,35,36]. The results showed that CgB could increase cell viability and mitochondrial membrane potential in a concentration-dependent manner, from 1 pM up to 10 nM, with the maximum effect observed at 10 nM. The effects of CgB also increased with stimulation time from 1 to 30 min.

In experiments regarding NO release, we found that CgB could increase it in a similar dose- and time-dependent manner as observed for cell viability and mitochondrial function. Additionally, CgB was able to counteract the effects of H_2_O_2_, improving cell viability and mitochondrial function and reducing NO release, all related to CgB concentrations from 1 pM to 10 nM. Notably, in the presence of CgB, ROS release was reduced, whereas GSH levels increased, with the effects of CgB increasing from 1 pM up to 10 nM. This data highlights the direct protective effect of CgB in HUVECs against peroxidative damage, potentially through the modulation of mitochondrial function. The latter finding is of relevance since it is well known that in endothelial cells, mitochondrial damage has widely been shown to represent an important factor in the onset of changes in vascular reactivity, which could lead to hypertension. In those processes, the loss of mitochondrial membrane potential and the increased mitoROS play a central role in the physiological regulation of vascular homeostasis. Indeed, changes in the membrane polarization could lead to alterations in the electron transport chain, which, in turn, may produce excessive ROS that would activate prothrombotic and proinflammatory pathways and reduce NO bioavailability. In this way, the balance between relaxing and constricting factors in endothelial cells would be lost, shifting towards increased vascular resistance. All the above factors, associated with the mitoROS-induced endothelial–mesenchymal transition, senescence, and atherosclerosis development, could lead to arterial hypertension [37]. Further research could focus on analyzing the levels of Peroxiredoxins, which have a significant role in protecting the cell and sensing oxidative stress during alterations of redox balance [38], to confirm the antioxidant actions of CgB and deepen the information about its protective effects on mitochondrial function.

Our results revealed the dual role of CgB in regulating NO release. Under physiological conditions, CgB increased NO release by HUVECs, whereas under oxidative stress, CgB inhibited NO release caused by H_2_O_2_. It is noted that we found similar opposite effects on NO release in HUVECs or other endothelial cells treated with other agents such as human chorionic gonadotropin, asenapine, artemetin, aflibercept, and ranibizumab [39].

These findings are particularly relevant considering the central role of mitochondria, redox state balance, and NO in maintaining vascular endothelial function. Endothelial dysfunction, a common early event in vascular diseases, can arise from mitochondrial damage [24]. In endothelial cells, unbalanced ROS generation, not countered by ROS-scavenging antioxidant systems, is considered a major risk factor for the development and progression of atherosclerosis [40]. Increased ROS production can reduce NO bioavailability, leading to arterial vasoconstriction and contributing to the development of hypertension [16,17]. The link between vascular inflammation, oxidative stress, pathological arterial changes, and increased blood pressure is well accepted. Endothelial dysfunction, recognized as a crucial factor in hypertension, involves NO production essential for maintaining vascular tone [22]. The dual role of NO in the cardiovascular system, determined by its production rate, coexistence of inflammation/oxidative stress conditions, and subtype of NOS, is widely acknowledged. Low levels of NO, such as those produced by endothelial NOS (eNOS), can protect against endothelial damage, while high concentrations, such as those produced by inducible NOS (iNOS) under oxidative stress conditions, can be harmful, leading to peroxynitrite formation [41].

Given the central role played by NO in the regulation of endothelial function, here we aimed to evaluate the effects of CgB on NO release in HUVECs. In our study, CgB increased NO release in HUVECs cultured under physiological conditions and reduced it in the presence of H_2_O_2_. Although not well examined, these results suggest a differential action of CgB on HUVECs either cultured in physiological or peroxidative conditions and, in particular, on eNOS and iNOS.

Our data also indicated the involvement of PKA, AMPK, Pi3K/Akt, MEK1/2/ERK1/2, and CaMKII pathways in the effects of CgB on HUVECs. Notably, these signaling pathways were implicated not only in NO release by modulating eNOS activity and calcium movements but also in cell survival, as with Pi3K/Akt and MEK1/2/ERK1/2 [20,21,22,23,24]. Furthermore, the augmented effects of the β-adrenergic receptor agonist isoproterenol on NO release, along with the abolition of NO release by the β_2_-adrenergic receptor antagonist butoxamine, confirmed the specific role of this receptor subtype in the response to CgB.

Analysis of Ca^2+^ movements induced by CgB in HUVECs cultured under physiological conditions revealed dose-response effects, with the highest response observed at CgB concentrations from 1 pM to 10 nM. In HUVECs cultured under peroxidative conditions, CgB dose-dependently reduced the increased [Ca^2+^]c caused by H_2_O_2_. Notably, the response of HUVECs to CgB was nearly abolished by EGTA, indicating that the mobilization of Ca^2+^ by CgB originated from the extracellular pool. Furthermore, experiments performed in the presence of H89, KN93, isoproterenol, and butoxamine showed the involvement of PKA, CaMKII pathways, and β_2_-adrenergic receptors in the effects of CgB on [Ca^2+^]c. These findings are consistent with previous observations regarding the role of these receptors and intracellular pathways in modulating Ca^2+^ transients in endothelial cells [24,42,43].

Overall, our results provide valuable insights into the effects of CgB on endothelial cells and the intracellular signaling pathways involved. In particular, the comparison between data we obtained and previous ones regarding the vascular/endothelial effects of members of the chromogranin family would highlight the involvement of common intracellular pathways, such as Pi3K/Akt and MEK1/2, β-adrenergic receptors, and NO release.

Concerning the latter issue, we highlighted the fact that our findings add information about the mechanisms involved in the response of endothelial cells to CgB. Hence, it could be hypothesized that under physiological conditions, CgB may activate eNOS through PKA, CaMKII, MEK1/2/ERK1/2, Pi3K/Akt, and by increasing [Ca^2+^]c. Under peroxidative conditions, CgB may reduce eNOS activation by modulating calcium movements. Additionally, the reduction of [Ca^2+^]c by CgB in HUVECs treated with H_2_O_2_ may represent one possible mechanism underlying the protective effects observed on mitochondrial function and ROS release. Finally, in peroxidative conditions, CgB could reduce iNOS. By acting on eNOS and iNOS, CgB would hinder the increased release of NO caused by H_2_O_2_ in HUVECs.

In-depth studies using immunofluorescence/Western blot in the presence or absence of β_2_-adrenergic receptor blocker could further elucidate the roles of the aforementioned kinases and the various NOS isoforms, and the quantification of cGMP levels could be useful to better characterize the role of CgB in the regulation of endothelial function under physiological and pathological conditions. Additionally, further research performed in endothelial cells could be focused on the evaluation of its effects on proliferation and migration, as well as understanding the relationship between CgB and catecholamines in NO release and the related involvement of calcium movements, β_2_-adrenergic receptors, and the Pi3K/Akt/MEK1/2/ERK1/2 pathways. Moreover, examining the role of NO in the beneficial effects of CgB in HUVECs against oxidative stress would be particularly interesting.

It should be highlighted that, in addition to increasing our knowledge about the role of CgB in endothelial cells, albeit with the limitations mentioned above, our findings could have significant clinical implications. While under physiological conditions, CgB contributes to maintaining vascular endothelial function under oxidative stress, which is observed in cases of cardiac dysfunction, coronary artery disease, and arterial hypertension; it protects the vasculature against peroxidation. Also, the modulation of endothelial function and Ca^2+^ movements could represent possible mechanisms underlying the antihypertensive effects of CgB, consistent with findings by Bartolumucci et al., who demonstrated an inverse relationship between CgB levels and arterial blood pressure [3].

## 4. Materials and Methods

### 4.1. Cell Culture

HUVECs (ATCC, catalog. no. CRL-1730TM) were cultured in Dulbecco’s Modified Eagle Medium (DMEM, Euroclone, Pero, Milan, Italy) containing 2 mM of L-glutamine (Euroclone), 1500 mg/L of sodium bicarbonate (Euroclone) supplemented with 0.1 mg/mL of heparin (Merck, Milan, Italy), 1% penicillin, 1% streptomycin, and 10% FBS (Euroclone). Cultures were maintained at 37 °C with 5% CO_2_. For all the assays, 1 × 10^4^ cells were plated in 96-well plates with DMEM 0% FBS supplemented with 1% penicillin–streptomycin–glutamine without phenol red (starvation medium, Sigma, Burlington, MA, USA) for 4–6 h. All the experiments were repeated three times on different pools of HUVECs, and the readings were performed in triplicate.

### 4.2. Cell Viability Measurement

Cell viability was examined in HUVECs by using the 1% 3-[4,5-dimethylthiazol-2-yl]-2,5-diphenyl tetrazolium bromide (MTT; Life Technologies Italia, Monza, Italy) dye, as previously executed [44,45,46,47].

In the preliminary phase, in HUVECs, we performed a grading and time-course study in order to establish the proper concentration and timing of stimulation for CgB, which were used to execute the experiments of the next phase performed in the presence of inhibitors. To do this, HUVECs were stimulated with fully synthetic human CgB (secretogranin I, 430–437) (catalog number 066-07; Phoenix Pharmaceutics; Burlingame, CA, USA) at 1 pM, 100 pM, 10 nM, and 100 nM for 1 min, 5 min, 15 min, 30 min, and 60 min. Experiments were repeated in the presence of H_2_O_2_ (200 μM, 30 min) and N-acetylcysteine (NAC; 200 μM, 30 min, Sigma), which was used as a positive control. In this case, 1 pM, 100 pM, 10 nM, and 100 nM of CgB were used at the timing selected in the preliminary phase.

In the experiments of the next phase, CgB at the selected concentration and timing was administered in the absence or presence of PKA, AMPK, pan-Pi3K inhibitor, MEK1/2, CaMKII inhibitors, namely, H89 (10 nM; Santa Cruz Biotechnologies, Dallas, TX, USA), dorsomorphin (10 nM; Sigma), wortmannin (10 nM; Sigma), UOI26 (10 nM; Santa Cruz Biotechnologies), and KN93 (10 nM; Sigma), dissolved in DMSO. Those inhibitors were given in costimulation with CgB.

After each stimulation, the medium was replaced with fresh culture medium (0% red phenol and 0% FBS). Then, the MTT dye was added to the well plates and left to incubate for 2 h at 37 °C. Thereafter, the medium was replaced with an MTT solubilization solution (dimethyl sulfoxide; Sigma) and mixed in order to dissolve the formazan crystals. In each sample, the absorbance was read at 570 nm through a spectrophotometer (VICTOR™ X Multilabel Plate Reader; Waltham, MA, USA). The viability of HUVECs was compared with that of control cells (non-treated cells; only DMSO), which was normalized to 100.

### 4.3. NO Release Measurement

The NO production was measured in HUVECs supernatants by using the Griess assay (Promega, Milan, Italy), as previously executed [44,45,46,47].

Also, regarding the NO release, we performed a preliminary phase, which was focused on the evaluation of the proper concentration and timing of stimulation for CgB. Some experiments were executed in the presence of H_2_O_2,_ as well. The experimental protocols were the same followed for cell viability. In addition, in the next phase, CgB at the selected concentration and timing, was administered in the absence or presence of the β-adrenergic receptor agonist, isoproterenol (10 nM; Sigma), the β_2_-adrenergic receptor antagonist, butoxamine (10 nM; Sigma, dissolved in DMSO), the α-adrenergic receptor agonist, phenylephrine (10 nM; Sigma, dissolved in DMSO) and antagonist, phentolamine (10 nM; Sigma, dissolved in DMSO), the muscarinic receptor antagonist, atropine (10 nM; Sigma, dissolved in DMSO), the NO synthase antagonist, Nω-Nitro-L-arginine methyl ester hydrochloride (10 mM; L-NAME, Sigma, dissolved in DMSO) in addition to H89, dorsomorphin, wortmannin, UOI26, and KN93. The above agonists/antagonists and inhibitors were given in costimulation with CgB. Acetylcholine (10 μM; Sigma, dissolved in DMSO) was used as a positive control.

After each stimulation, NO production in the sample supernatants was examined by adding an equal volume of Griess reagent following the manufacturer’s instruction. Briefly, sulfanilamide solution and NED solution were allowed to equilibrate to room temperature for 15–30 min. Thereafter, 50 μL of each sample was added to the wells in triplicate. By using a multichannel pipettor, 50 μL of the sulfanilamide solution was added to all experimental samples. After 10 min incubation at room temperature in the dark, 50 μL of the NED solution was dispensed to all wells. The absorbance at 570 nM was measured by a spectrometer (VICTOR™ X Multilabel Plate Reader), and the NO production was quantified according to the nitrate standard curve. The values obtained corresponded to the NO (µM) produced after each stimulation by samples containing 1.5 µg of protein each. The NO release from HUVECs was compared with that of control cells (non-treated cells; only DMSO), which was normalized to 1.

### 4.4. Mitochondrial Membrane Potential Measurement

We used the JC-1 assay in order to examine the mitochondrial membrane potential of HUVECs, as we did in previous studies [46,47].

HUVECs were stimulated with 1 pM, 100 pM, 10 nM, and 100 nM of CgB at the timing selected in the preliminary phase, in the presence or absence of H_2_O_2_ (200 μM, 30 min), as performed for cell viability and NO release. Moreover, in the next phase, CgB at the selected concentration and timing was administered in the absence or presence of H89, dorsomorphin, wortmannin, UOI26, and KN93 (10 nM, dissolved in DMSO, costimulation with CgB; Sigma). After each stimulation, the medium was removed, and cells were incubated for 15 min at 37 °C with the 5,51,6,61-tetrachloro-1,11,3,31 tetraethylbenzimidazolyl carbocyanine iodide (JC-1) 1× diluted in Assay Buffer 1× (Cayman Chemical, Ann Arbor, MI, USA). After washing twice using the Assay Buffer 1×, the red (excitation 550 nm/emission 600 nm) and green (excitation 485 nm/emission 535 nm) fluorescence was read using a spectrophotometer (VICTOR™ X Multilabel Plate Reader). The mitochondrial membrane potential of HUVECs was compared with that of control cells (non-treated cells; only DMSO), which was normalized to 100.

### 4.5. ROS Release Measurement

The oxidation of 2,7-dichlorodihydrofluorescein diacetate (H2DCFDA) into 2,7-dichlorodihydrofluorescein (DCF) was used to assess ROS release, following the manufacturer’s instructions (Abcam; Cambridge, UK), and as previously performed [44,45,48,49].

HUVECs were stimulated with 1 pM, 100 pM, 10 nM, and 100 nM of CgB at the timing selected in the preliminary phase, in the presence of H_2_O_2_ (200 μM, 30 min). NAC, at 200 μM and 30 min stimulation, was used as a positive control. After each simulation, staining was performed with 10 μM of H2DCFDA for 20 min at 37 °C. The fluorescence intensity of DCF was measured at an excitation and emission wavelength of 485 and 530 nm, respectively, by using a spectrophotometer (VICTOR™ X Multilabel Plate Reader). Results were expressed as DCF fluorescence intensity.

### 4.6. GSH Measurement

In HUVECs, GSH measurements were performed with a specific kit (Cayman Chemical), as previously described [50,51]. HUVECs were treated as executed for ROS release, and after the treatments, the cells were lysed using 50 mM of 2-(N-morpholino) ethanesulphonic acid (GSH MES Buffer) and a rubber policeman. The cell pellet was centrifuged at 10,000× *g* for 15 min at 4 °C. After centrifugation, the supernatant was treated with an equal volume of metaphosphoric acid (final concentration 5%; Sigma) for 5 min and centrifuged at 2000× *g* for at ≥2 min. The supernatant was collected and supplemented with 50 µL per ml of a 4 M solution of triethanolamine (Sigma). Then, 50 µL of the samples were transferred to a 96-well plate where GSH was detected following the manufacturer’s instructions using a spectrometer (VICTOR™ X Multilabel Plate Reader) at excitation/emission wavelengths of 405–414 nM. GSH was expressed as nanomoles in samples with 1.5 mg of protein/mL.

### 4.7. Measurement of [Ca^2+^]c by Fura-2 Fluorescence

To measure [Ca^2+^]c, HUVECs were grown to confluence, washed twice with sterile phosphate buffer saline 1× (Euroclone), and incubated with 5 μM of fura-2/acetoxymethyl (AM) ester (Sigma) in DMEM (Euroclone) containing 10% FBS and without phenol red for 30 min in the dark. After additional washings with DMEM (Euroclone), the coverslips were mounted in a thermostatted quartz cuvette and placed in an agitation system at 37 °C. The measurement was performed using a Hitachi F-4500 Fluorescence Spectrometer (Hitachi High-Technologies Corporation, Tokyo, Japan) for a continuous duration of 300 s at an excitation wavelength of 340 nm and an emission wavelength of 510 nm.

In the preliminary phase, Fura-2/AM-loaded HUVECs were stimulated with CgB at 1 pM, 100 pM, 10 nM, and 100 nM at the timing of stimulation selected in HUVECs, in the presence or absence of H_2_O_2_ (200 μM, 30 min). The effects of CgB were compared with those elicited by ATP (10 μM; Sigma). In addition, some experiments were performed in the presence or absence of Ca^2+^ in the incubation medium, which was obtained with 50 mM of ethylene glycol tetraacetic acid (EGTA, Sigma). Also, in some experiments, CgB at the concentration and timing selected in the preliminary phase was administrated in the presence or absence of isoproterenol, butoxamine, H89, and KN93 (10 nM, dissolved in DMSO, costimulation with CgB).

The quantification of [Ca^2+^]c was executed using the following equation, as previously reported [24,47,52,53]: (Ca^2+^) = Kd ((R − Rmin)/(Rmax − R)). The Kd of fura-2/AM for Ca^2+^ was considered 224. R_min_ and R_max_ were the minimum and maximum values of fluorescence ratio obtained under Ca^2+^ free (EGTA 0.1 M) or Ca^2+^ saturated conditions, respectively. The fluorescence intensities obtained were corrected for cell autofluorescence at the respective wavelengths employed.

### 4.8. Statistical Analysis

All data are presented as the median and range of three different experiments performed on different pools of HUVECs and triple readings. The differences between two or more groups were assessed through the Mann–Whitney test and Kruskal–Wallis test, followed by Dunn’s post hoc test for multiple comparisons, respectively. The non-parametric Mann–Kendall trend test was used to evaluate the trend of values over time. A value of *p* < 0.05 was considered statistically significant. Statistical analysis was performed, and graphs were made using GraphPad Prism version 9.0.0 (GraphPad Software; San Diego, CA, USA) and STATA v.17 (StataCorp. 2021 Statistical Software: Release 17; College Station, TX, USA).

## 5. Conclusions

The data obtained from this study underscore the beneficial effects exerted by CgB in HUVECs, including the maintenance of endothelial function under physiological conditions and the mitigation of oxidative stress effects. Based on our findings, it could be speculated that increased CgB release observed in pathological conditions might counteract the effects induced by catecholamines, as demonstrated in our HUVEC model. In this way, CgB could act as a modulatory agent in the cardiovascular system and be considered a central factor in preventing cardiovascular diseases through its direct actions on endothelial cells. To further elucidate these mechanisms, additional studies will be, however, necessary to clarify the effects of CgB on NOS isoforms, the kinases involvement, the relationship with β_2_-adrenergic receptors and calcium movements, and to evaluate the response of other cell lines, such as smooth muscle cells, to CgB.

## Data Availability

The data that support the findings of the present study are available from the corresponding author upon reasonable request.

## Supplementary Materials

The following supporting information can be downloaded at: https://www.mdpi.com/article/10.3390/ijms251910296/s1.
