# Supplementary material for: Chromogranin B Protects Human Umbilical Endothelial Cells against Oxidative Stress
Source: Int J Mol Sci. 2024 Sep 25;25(19):10296. doi: 10.3390/ijms251910296 (PMC11476595; doi:10.3390/ijms251910296)
Supplement: Supplementary file 1 [file ijms-25-10296-s001.zip › ijms-3081042-supplementary.pdf]

Supplemental figures

A

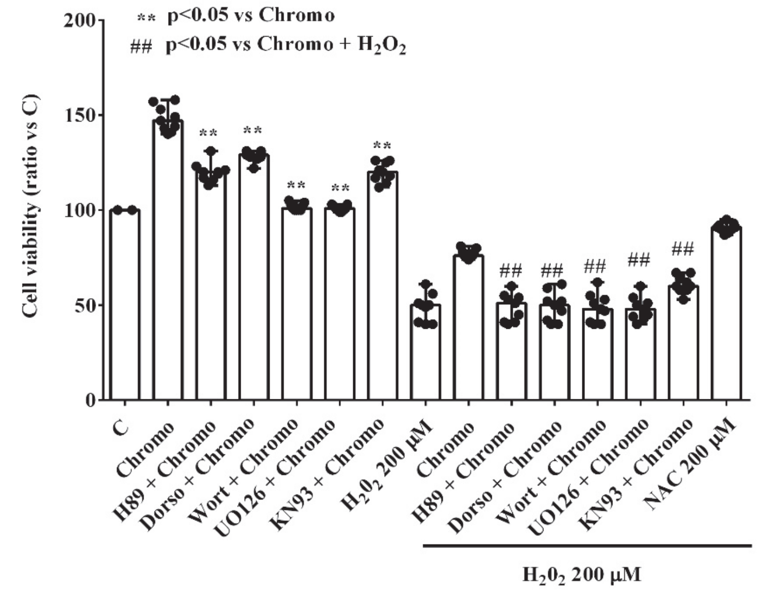

B

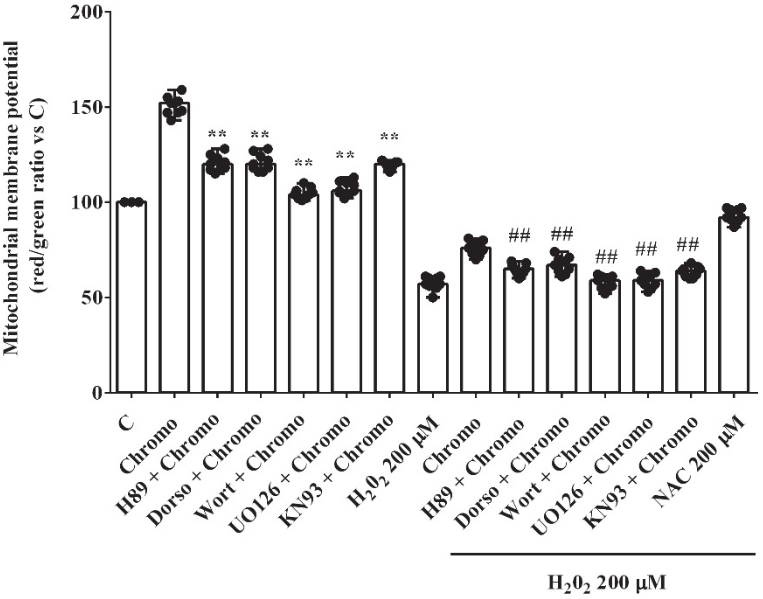

Figure S4. Effects of 10 nM chromogranin B on cell viability (A) and mitochondrial membrane potential (B) in HUVEC in the presence or absence of various agonist/antagonists. The results are the medians and range of experiments repeated three times on different pools of HUVEC and triple readings (n=9). C= untreated cells. Chromo= chromogranin B. Dorso=dorsomorphin (10 nM). H89= PKA inhibitor (10 nM). KN93= CAMKII inhibitor (10 nM). NAC = N-acetylcysteine. UO126= MEK1/2 inhibitor (10 nM). Wort= wortmannin (pan-PI3K inhibitor) (10 nM). The Mann-Whitney test was used to compare the results of three different experiments performed for each experimental setting (Chromo with various inhibitors with and without H_2O_2) with the reference group (Chromo alone or with H_2O_2). ** indicates statistical significance vs Chromo. ## indicates statistical significance vs Chromo + H_2O_2. A *p* value <0.05 was chosen for statistical significance.

A

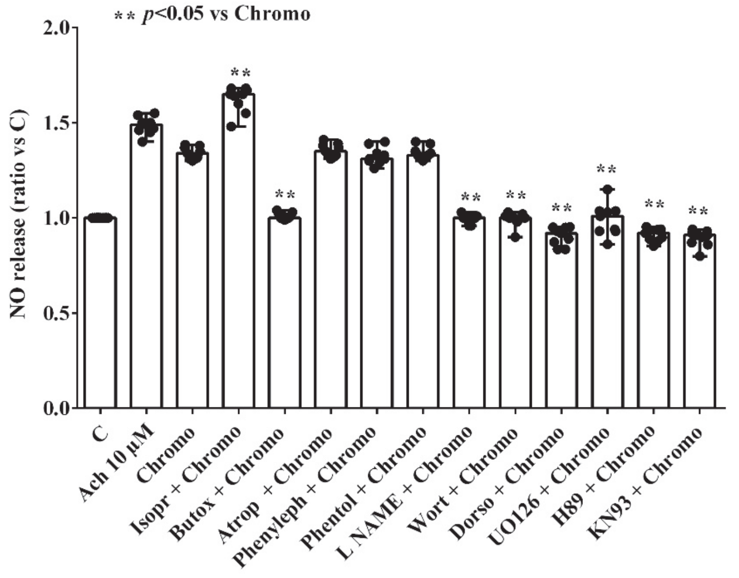

B

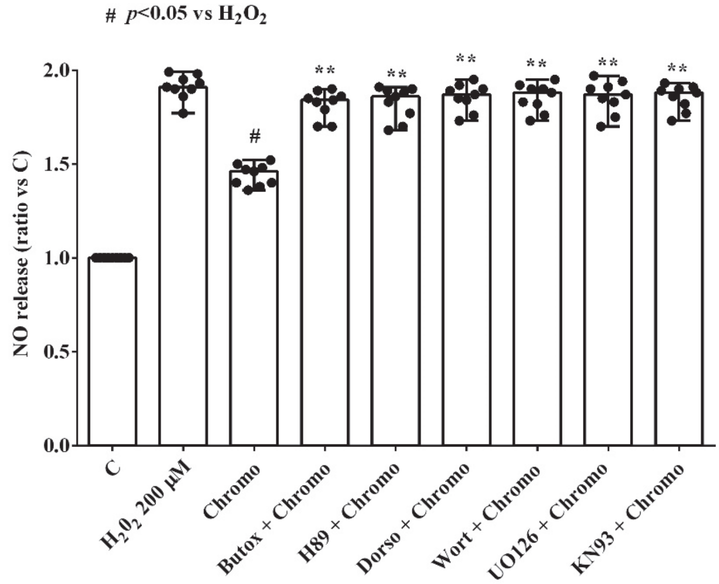

Figure S5. Effects of 10 nM chromogranin B on nitric oxide (NO) release in HUVEC in the presence or absence of various agonists/antagonists. The results are the medians and range of experiments repeated three times on different pools of HUVEC and triple readings (n=9). Ach= acetylcholine. Atrop= atropine (cholinergic receptors inhibitor; 10 nM). Butox= butoxamine (β_2 adrenergic receptors inhibitor; 10 nM). Isopr= isoproterenol (β adrenergic receptors agonist; 10 nM). L-NAME= Nω-Nitro-L-arginine methyl ester hydrochloride (NO inhibitor; 10 mM). Phenyleph= phenylephrine (α adrenergic receptors agonist; 10 nM). Phentol= phentolamine (α adrenergic receptors antagonist; 10 nM). Other abbreviations are as in the previous figure. The Mann-Whitney test was used to compare the results of three different experiments performed for each experimental setting (Chromo with various inhibitors/agonist with and without H_2O_2) with the reference group (Chromo alone or with H_2O_2). ** indicates statistical significance vs Chromo. A p value <0.05 was chosen for statistical significance.

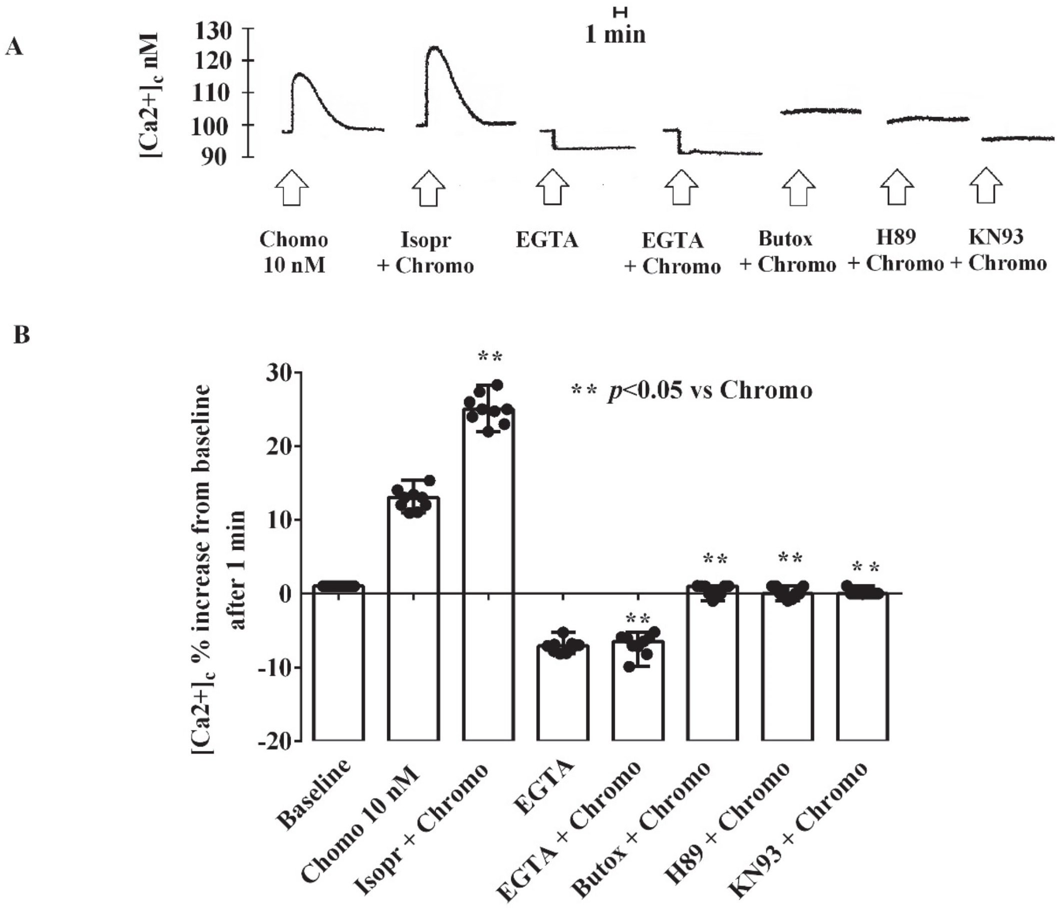

Figure S7. Effects of chromogranin B 10 nM on [Ca^{2+}]c in HUVEC cultured in physiologic conditions in the presence or absence of various agonist/antagonists and ethylene glycol tetra-acetic acid (EGTA). In A, an example of traces is shown; in B, results obtained from three different experiments performed on different pools of HUVEC and triple readings (n=9) as medians and range. EGTA= ethylene glycol tetraacetic acid (50 mM). Other abbreviations are as in the previous figures. The Mann-Whitney test was used to compare the results of three different experiments performed for each experimental setting (Chromo with various inhibitors/EGTA/agonist) with the reference group (Chromo). ** indicates statistical significance vs Chromo. A *p* value <0.05 was chosen for statistical significance.
